# Outer Membrane Vesicle Induction and Isolation for Vaccine Development

**DOI:** 10.3389/fmicb.2021.629090

**Published:** 2021-02-04

**Authors:** Melanie D. Balhuizen, Edwin J. A. Veldhuizen, Henk P. Haagsman

**Affiliations:** Division of Infectious Diseases and Immunology, Department of Biomolecular Health Sciences, Faculty of Veterinary Medicine, Utrecht University, Utrecht, Netherlands

**Keywords:** outer membrane vesicles, isolation, induction, vaccine development, *Bordetella pertussis*, *Neisseria meningitidis*, host defense peptides

## Abstract

Gram-negative bacteria release vesicular structures from their outer membrane, so called outer membrane vesicles (OMVs). OMVs have a variety of functions such as waste disposal, communication, and antigen or toxin delivery. These vesicles are the promising structures for vaccine development since OMVs carry many surface antigens that are identical to the bacterial surface. However, isolation is often difficult and results in low yields. Several methods to enhance OMV yield exist, but these do affect the resulting OMVs. In this review, our current knowledge about OMVs will be presented. Different methods to induce OMVs will be reviewed and their advantages and disadvantages will be discussed. The effects of the induction and isolation methods used in several immunological studies on OMVs will be compared. Finally, the challenges for OMV-based vaccine development will be examined and one example of a successful OMV-based vaccine will be presented.

## Introduction on Outer Membrane Vesicles

**Figure 1:**
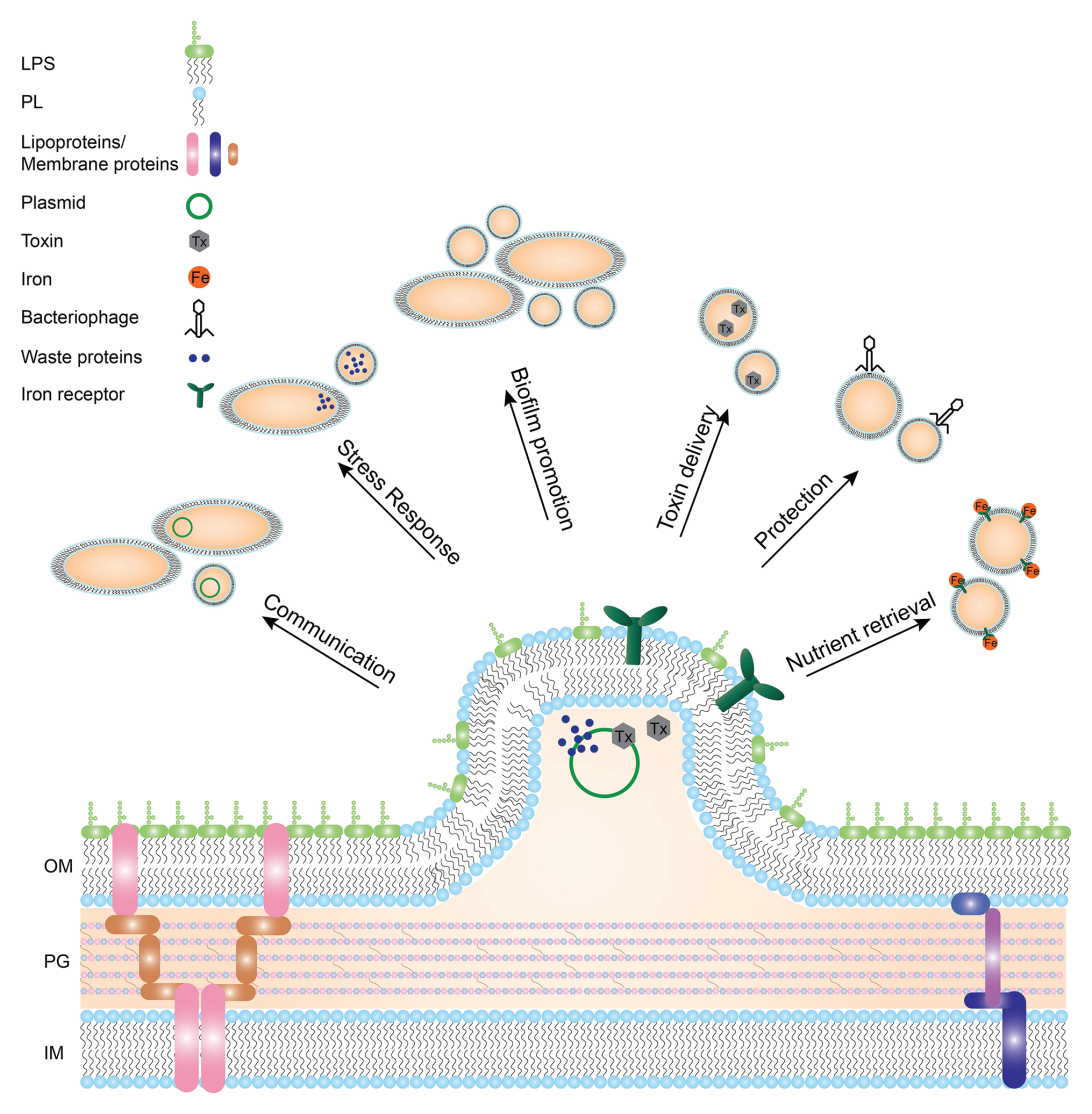
Gram-negative bacterial membrane during OMV formation and functions of resulting OMVs. OMVs have been implicated in many different processes. Depicted here are the different functions OMVs have been shown to be involved in such as transport of toxins, waste removal, or communication between bacteria. LPS: lipopolysaccharide, PL: phospholipid, OM: outer membrane, PG: peptidoglycan, IM: inner membrane.

Gram-negative bacteria have two membranes, the inner membrane (IM) and the outer membrane (OM) with a network of peptidoglycan (PG) and the periplasmic space in between. Both the IM and OM consist of phospholipids and membrane proteins, with only the outer leaflet of the OM containing lipopolysaccharide (LPS). From the OM, small protrusions can form that pinch off and become extracellular vesicles, called outer membrane vesicles (OMVs; Figure 1; Beveridge, 1999). Resulting OMVs are between 20 and 300 nm in diameter. They consist of a single lipid bilayer containing LPS, phospholipids, and various outer membrane proteins (OMPs), which represents the OM of the originating bacteria. Formation of OMVs has been the subject of much debate, since the driving force of OMV formation was long unknown (Zhou et al., 1998; Haurat et al., 2011). The formation of OMVs was long thought to be an arbitrary stress response from bacterial cells (McBroom and Kuehn, 2007), but OMVs were later proven to have many more functions, which will be discussed below.

### Formation of OMVs

For OMV formation, it is necessary to detach the OM from the PG layer and the IM. These layers are stably linked by many different lipoproteins. A local decrease in the number of lipoproteins, and therefore the number of crosslinks, has been implicated in OMV formation. For example, the deletion of lipoprotein (Lpp) in combination with magnesium starvation or the deletion of outer membrane protein A (OmpA) in *Escherichia coli* that results in hypervesiculating mutants (Sonntag et al., 1978; Yem and Wu, 1978). Similarly, the Tol-Pal system consists of several proteins connecting the IM with the OM and disruption of the Tol-Pal system resulted in hypervesiculation in *Salmonella* and *E. coli* (Bernadac et al., 1998; Deatherage et al., 2009). Furthermore, alterations to the PG structure can prevent proper attachment of lipoproteins, which in turn decreases the number of crosslinks between the IM and OM. This indirectly causes an increase in OMV formation due to outer-membrane instability. For instance, a PG hydrolase mutant of *E. coli*, defective in peptide crosslinks of the PG, prevented attachment of Lpp in the PG layer (Schwechheimer et al., 2014).

An increase in membrane turgor, the force of internal fluids pressing outward, also results in an increase in OMV release. For example, the accumulation of misfolded periplasmic proteins in a periplasmic serine endoprotease (*degP*) mutant, resulting in loss of a periplasmic chaperone, resulted in hypervesiculation (Schwechheimer and Kuehn, 2013). In a mutant defective in PG recycling, PG fragments accumulated and increased membrane turgor, leading to membrane pressure and increased OMV release. It was shown that Lpp-based crosslinks between the PG and IM in this mutant remained at a similar level as in the wild-type strain (Schwechheimer et al., 2014). This suggests that this mechanism is independent of crosslink formation and therefore increases OMV formation through a distinct mechanism (Schwechheimer and Kuehn, 2013; Schwechheimer et al., 2014).

The most recent hypothesis for OMV formation is the induction of curvature in the OM due to an increase in phospholipid (PL) content. An increase in OMV formation was shown for mutants missing components of a retrograde PL transporter system (Roier et al., 2016). This was shown for *Haemophilus influenzae*, *Vibrio cholerae*, and *E. coli*, indicating that it is a conserved mechanism in several species. Altogether, many different mechanisms for OMV formation have been described in the past decades and most likely all these mechanisms are simultaneously at play in bacteria (McBroom et al., 2006; Kulp and Kuehn, 2010; Kulkarni and Jagannadham, 2014; Schwechheimer and Kuehn, 2015).

### Functions of OMVs

OMVs exert many different functions, all beneficial to the bacterium. Mostly, OMVs act as a transportation system not only for proteins, but also for DNA and RNA (Dorward et al., 1989; Koeppen et al., 2016; Bitto et al., 2017). Vesicles provide a protected environment for bacterial molecules and delivery by OMVs may act as a long-distance delivery system (Bomberger et al., 2009; Jones et al., 2020). Additionally, transport by OMVs prevents dilution of cargo. OMV cargo has been shown to be involved in inter-cellular communication. For example, OMVs of *Pseudomonas aeruginosa* contain the Pseudomonas quinolone signal (PQS) and removal of OMVs from the bacterial culture inhibits cell-cell communication (Mashburn and Whiteley, 2005). Furthermore, antibiotic resistance genes are often transported *via* OMVs. OMVs from *Neisseria gonorrhoeae* were shown to contain circular DNA and supplementation with these OMVs provided penicillin resistance in susceptible bacterial strains (Dorward et al., 1989). Additionally, *Acinetobacter baumannii* was shown to transfer carbapenem resistance genes in their OMVs (Rumbo et al., 2011). However, OMVs are not only used for communication within one bacterial species, but also for inter-species communication. When *E. coli* or *Salmonella* species were incubated with OMVs derived from *P. aeruginosa* or *Shigella flexneri*, antigens of the latter two were readily detected on the surface of the first two bacterial species, suggesting inter-species communication by OMVs (Kadurugamuwat and Beveridge, 1999). Furthermore, *E. coli* OMVs were shown to package Shiga toxins (Kolling and Matthews, 1999) and *P. aeruginosa* OMVs were shown to contain PG hydrolases and fuse with *E. coli* and *Staphylococcus aureus* membranes, thereby eradicating competing bacterial species (Kadurugamuwa and Beveridge, 1996).

Besides bacterial interactions, OMVs are involved in pathogen-host interactions (Jones et al., 2020; Zingl et al., 2020). OMVs are used by many bacterial species to deliver toxins and other virulence factors (Kadurugamuwa and Beveridge, 1995; Ellis and Kuehn, 2010; Yoon, 2016; Gasperini et al., 2017; Jha et al., 2017). For example, *P. aeruginosa* was shown to package small RNAs in OMVs that silenced host RNA involved in the innate immune response (Koeppen et al., 2016). Sorting of OMV cargo must therefore be a selective process and might be regulated by LPS microdomains, but the exact sorting mechanism has yet to be elucidated (Haurat et al., 2011; Bonnington and Kuehn, 2014).

Furthermore, OMVs are beneficial to bacterial growth in several ways. Despite the fact that OMV release seems to be a one-way process, OMVs also have been shown to fuse with bacterial membranes, for instance to aid in nutrient acquisition. For *Neisseria meningitidis*, it was shown that OMVs are enriched in proteins involved in iron and zinc acquisition (Lappann et al., 2013). Similarly, for *Bordetella pertussis*, the process of iron retrieval by OMVs from medium was demonstrated. When OMVs from an iron-rich culture were supplemented to a culture growing in iron-limited conditions, they were able to transfer iron to bacterial cells and boost bacterial growth (Gasperini et al., 2017).

Another function related to OMV production is protection, both from exogenous and endogenous molecules. For instance, OMVs are used to dispose of bacterial waste, such as misfolded proteins, to prevent bacteria from collapsing under the pressure (Manning and Kuehn, 2013; Schwechheimer et al., 2013). This is regulated by stress responses, such as the sigma E pathway (Kulp and Kuehn, 2010) or independent of envelope stress responses (McBroom and Kuehn, 2007), as a protection mechanism. Many exogenous molecules can also threaten bacteria such as antimicrobial peptides (AMPs) and bacteriophages. Addition of OMVs to an *E. coli* or *Helicobacter pylori* culture increased bacterial resistance to AMPs and bacteriophages (Manning and Kuehn, 2011; Roszkowiak et al., 2019; Murray et al., 2020), presumably by acting as a decoy for these substances to attach to, instead of targeting the bacterial membrane.

The functions of OMVs in biofilms have been described in all stages of biofilm formation, being a common component of the biofilm matrix (Schooling and Beveridge, 2006). Addition of OMVs to *H. pylori* cultures was shown to correlate with increased biofilm forming ability (Yonezawa et al., 2009). OMVs of *P. aeruginosa* have been shown to aid in attachment and aggregation of bacterial cells in early stages of biofilm formation and carry molecules to protect the biofilm later on such as β-lactamases (Ciofu, 2000). The most well-known functions of OMVs are schematically depicted in Figure 1.

Despite the many physiological functions of OMV, their release is often insignificant and insufficient for industrial purposes (van der Pol et al., 2015). Several methods exist to induce OMV release in bacterial cultures and increase OMV yields (Klimentová and Stulík, 2015). However, these induced OMVs may have different properties compared to OMVs that are spontaneously released from bacteria (Collins, 2011; Schwechheimer and Kuehn, 2013; Michel et al., 2020; Balhuizen et al., 2021). In this review, we describe the different methods used to induce OMVs and to compare the properties of the resulting vesicles. Additionally, a standard nomenclature is introduced to prevent confusion between different types of OMVs. The potential of OMV-based vaccines is illustrated using *N. meningitidis* as an example, since it is the only licensed OMV-based vaccine to date. Furthermore, we compared immunological properties of differently induced OMVs from *B. pertussis*, a pathogen for which an OMV-based vaccine exhibits great potential. Future challenges for OMV-based vaccines are discussed, as well as different applications for use of OMVs.

## Induction and Isolation of OMVs for Therapeutic Purposes

OMVs have many potential therapeutic applications, which will be described later, but often their release is insignificant, resulting in low harvested yields from bacterial cultures. Spontaneous OMVs (sOMVs) are naturally released by Gram-negative bacteria and considered most similar to OMVs formed *in vivo* based on protein and lipid content (Gasperini et al., 2017, 2018; Valguarnera and Feldman, 2017). These OMVs can be obtained by growing bacteria until end-logarithmic phase and harvested without the addition of any foreign molecules. Therefore, all OMVs have been formed spontaneously and resemble the composition of *in vivo* formed OMVs by unstressed bacteria. The low yield of sOMVs makes them not easily feasible for vaccine production, yet these vesicles are most desirable for vaccine development due to their natural composition resembling the outer membrane of the bacterium.

### Induction Methods of OMVs

**Figure 2:**
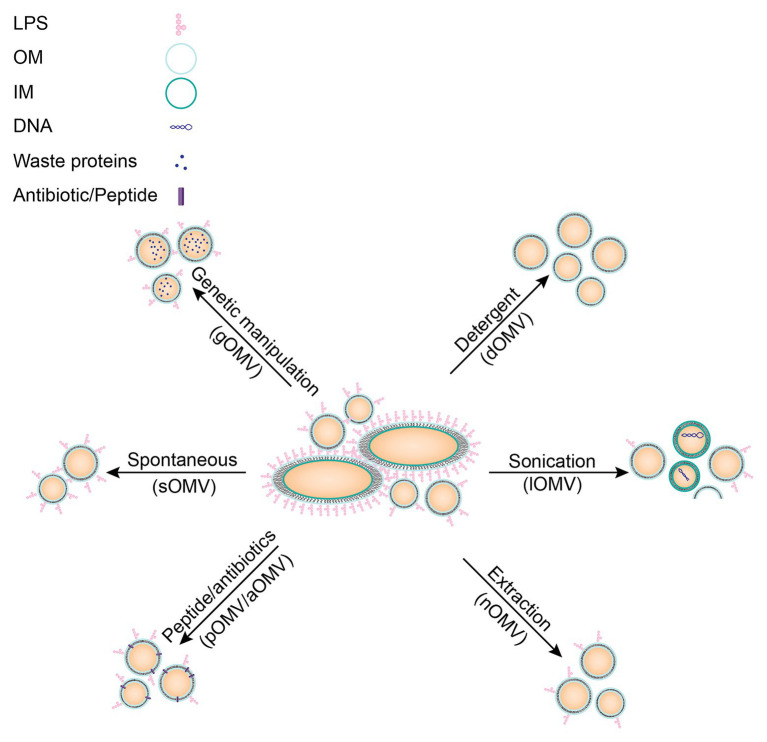
Schematic overview of different induction methods for OMVs including characteristics of resulting OMVs. No stimulation: these vesicles are most similar to spontaneous vesicles released *in vivo* (sOMVs). Genetic manipulation may alter OMV cargo (gOMVs). Detergent isolation of OMVs results in OMVs lacking LPS (dOMVs), an important immunogenic molecule. Sonication of bacteria disrupts the entire membrane, resulting in impurities in the vesicles’ fraction due to cell lysis (lOMVs). Extraction with membrane destabilizing molecules may alter vesicle composition (nOMVs), but they are more representative of the OM. OMV induction by peptides or antibiotics may alter membrane stability and may result in the peptide or antibiotic being present in the resulting OMV (pOMVs/aOMVs). A new technique researched to induce OMVs is heat-shock, resulting in hOMVs, but this technique is not yet established and therefore not included in this figure.

Several methods exist to increase release of OMVs, all with their own advantages and disadvantages (Figure 2). For instance, vesicles can be induced by disruption of the membrane with either addition of a detergent or by sonication. OMVs can also be induced by an extracting agent, such as ethylenediaminetetraacetic acid (EDTA), or with sub-lethal concentrations of antibiotics (Maredia et al., 2012; Duperthuy et al., 2013). Furthermore, OMVs might also be induced by genetic modifications, which will be discussed in more detail below. These different methods can all distinctly affect the resulting OMVs in size, proteolytic or thermal stability, or composition, which may influence the immune responses evoked by the OMVs (Collins, 2011).

In the next paragraphs, different methods to induce OMVs will be discussed in more detail, starting with genetic modifications that are applied to increase yields of OMVs (gOMVs; Ojima et al., 2020). Included in the term gOMVs are generalized modules for membrane antigens (GMMA), since this term likewise refers to OMVs from bacteria in which mutations induce hypervesiculation (Gerke et al., 2015). These gOMVs can be produced by various mutations, for example, by deletion of the *tolR* gene, which is part of the Tol-Pal system discussed above (Micoli et al., 2020). The Tol-Pal system has often been a target for creation of hypervesiculating mutants (Henry et al., 2004; Chen et al., 2016; Stevenson et al., 2018). Deletion of lipoproteins connecting the OM and the PG layer, such as Lpp for *E. coli*, has been shown to increase OMV production (Sonntag et al., 1978). Another example is the knock-out of chaperones to increase stress due to the presence of misfolded proteins, which in turn increases vesicle formation, as shown for a *degP* mutant of *E. coli* (McBroom et al., 2006). Deletion of a lytic transglycosylase, which resulted in hypervesiculating *N. meningitidis* strain, is another example of a long list of deletion mutants (Adu-Bobie et al., 2004; Ferrari et al., 2006). Deletion of genes is not the only modification that resulted in hypervesiculating bacteria. For example, overexpression of the outer membrane protease OmpT resulted in hypervesiculation in *E. coli* (Premjani et al., 2014). Additionally, expression of the deacylase PagL resulted in hypervesiculation, due to increased curvature of the bacterial outer membrane, caused by an inverted cone-shaped LPS (Elhenawy et al., 2016). This list is not exhaustive and research is still performed to identify additional hypervesiculating mutations. These modifications result in spontaneously formed vesicles, but a disadvantage is that these gOMVs can differ from *in vivo* formed vesicles since the bacterium has been genetically altered. For example, cargo in gOMVs resulting from a *degP* mutant is substantially different from cargo in sOMVs, with an increased presence of periplasmic proteins, which are suggested to be misfolded (Schwechheimer and Kuehn, 2013). Analysis of gOMVs produced by a *ΔtolB* mutant in *Buttiauxella agrestis* even revealed multilamellar vesicles (Takaki et al., 2020) and *E. coli* ΔtolR gOMVs were shown to have reduced entry into epithelial Caco-2 cells (Pérez-Cruz et al., 2016). Another disadvantage of genetic modification is that one mutation may not work in all Gram-negative bacteria, requiring research to find distinct mutations for different bacteria. To facilitate this, publishing data on genetic mutations not resulting in hypervesiculating bacterial strains would prevent other research groups from trying similar strategies.

A second method, using detergent for the extraction of OMVs (dOMVs), has been used for decades and is a widespread method in industry. *Neisseria meningitidis* OMV vaccines used to be prepared based on detergent extraction (Acevedo et al., 2014). With this method OMVs are induced with detergent-like molecules such as deoxycholate or sodium dodecyl sulfate. These molecules interact with the bacterial membrane to increase vesicle formation and additionally remove LPS from the outer membrane, creating LPS-containing micelles. The resulting dOMVs lack LPS (van der Pol et al., 2015), which will decrease the undesired LPS-based innate immune response. However, the loss of LPS results in loss of many antigens, which are loosely attached to the membrane. Additionally, the intrinsic adjuvant activity of OMVs is likewise lost upon LPS removal (van der Pol et al., 2015). This shows that there is a fine balance between potential beneficial and detrimental effects of LPS in OMVs.

Furthermore, OMVs can be induced by membrane destabilization using sonication, which does not remove LPS from the membrane (Roberts et al., 2008; Asensio et al., 2011; Bottero et al., 2018). These vesicles are prepared by sonication of the bacterial pellet, thereby forming membrane fragments, which fuse to form lysis OMVs (lOMVs). These lOMVs are not prepared from the bacterial supernatant, where the sOMVs can be found, and therefore likely contain cargo not natively present in OMVs (Roberts et al., 2008). So, despite high OMV yields obtained through sonication of bacteria, these vesicles do not represent *in vivo* protein compositions of sOMVs and therefore are not always suitable for vaccine development.

Another method to induce OMVs is the use of extraction molecules such as EDTA (van de Waterbeemd et al., 2010). These extraction molecules aim to destabilize the bacterial membrane similar to the two methods described above (dOMVs and lOMVs) but are relatively mild and thus retain LPS and native cargo in the OMVs (van de Waterbeemd et al., 2013a). Therefore, they are named native OMVs (nOMVs). One such a molecule is EDTA, which is a chelating agent that removes calcium ions from the environment (Hart, 2000). Calcium ions stabilize bacterial membranes by neutralization of repelling negative charges of LPS and other anionic lipids (Thomas and Rice, 2014). Removal of calcium ions causes the negative charges of LPS to repel each other, and thereby it destabilizes the membrane (Clifton et al., 2015). Therefore, yields of OMVs are increased using EDTA, but LPS remains present. These vesicles are better suited for vaccine development but might be less stable due to the lack of calcium ions.

Yet another method to increase OMV release is the induction of membrane stress by supplementation of external molecules, as was shown for naturally occurring antimicrobial peptides (AMPs; Manning and Kuehn, 2011; Balhuizen et al., 2021). These OMVs have been named peptide induced OMVs (pOMVs). AMPs are part of the innate immune system and are expressed by different cell types, such as granulocytes or epithelial cells, in response to bacterial signals, such as LPS, or cytokines, such as interleukin 1-beta (IL-1β; Zasloff, 2002; van Harten et al., 2018; Mookherjee et al., 2020; Scheenstra et al., 2020). These AMPs often have high affinity for bacterial membranes, which is part of their antibacterial mechanism of action (Zanetti, 2004; Hazlett and Wu, 2011; Schneider et al., 2016, 2017). As discussed above, increased OMV production may be a means of the bacterium to protect from induced stress. *Bordetella bronchiseptica* pOMVs resulting from induction by the porcine myeloid antimicrobial peptide 36 (PMAP-36) were indeed shown to contain PMAP-36 (Balhuizen et al., 2021). Furthermore, pOMVs contained relatively more phosphatidylglycerol compared to sOMVs, a negatively charged lipid, which might interact with the positively charged AMP. *Bordetella bronchiseptica* pOMVs were also shown to have decreased thermal stability compared to sOMVs, possibly due to the presence of PMAP-36 in the membrane (Balhuizen et al., 2021). As for peptide-based antibiotics, such as polymyxin B, also these molecules were shown to induce OMV release (Kulkarni et al., 2015). The mechanism of OMV induction might be similar to AMPs mechanism and is based on membrane disruption resulting in stress for the bacterium and subsequent OMV production.

Antibiotics targeting intracellular processes were also shown to induce OMV release. OMV formation might be a response to antibiotics, since in *P. aeruginosa* it was shown that antibiotics induce PQS secretion (Bru et al., 2019) and PQS was shown to induce OMV formation (Schertzer and Whiteley, 2012). However, antibiotic-induced OMVs (aOMVs) are mostly characterized based on protein content (Godlewska, 2019), so a good comparison to pOMVs cannot be made yet. aOMVs of extra-intestinal pathogenic *E. coli* were characterized after induction by gentamicin and particle sizes were not altered (Chan et al., 2017). Remarkably, when the cargo of these aOMVs was assessed using mass-spectrometry, mostly cytoplasmic and periplasmic proteins were enriched, relative to sOMVs. Likely these are misfolded proteins formed after gentamicin’s interference with the ribosome machinery. *Escherichia coli* aOMVs induced by ampicillin were shown to have an increased amount of the OMP Pal, further demonstrating that antibiotics can alter OMV cargo (Michel et al., 2020). In another study, *Acinetobacter baumannii*, was stimulated with tetracycline, imipenem, and eravacycline, and the resulting OMVs were quantified (Yun et al., 2018; Kesavan et al., 2020). Whereas tetracycline did not induce OMV release, imipenem did induce release of aOMVs, which showed a relative increase in OMPs and proteases (Yun et al., 2018). Eravacycline-induced aOMVs likewise contained not only relatively more OMPs but also resistance-associated proteins such as ATP-binding cassette (ABC) and other transporter proteins (Kesavan et al., 2020). This demonstrates the possible risks of this induction method, as sub-lethal concentrations of antibiotics may result in development of antibiotic resistance.

Since these different methods all result in slightly different vesicles, nomenclature to distinguish between different categories is important. However, the current use of abbreviations in literature is not consistent. Different abbreviations are used in literature for an identical OMV type while, vice versa, one abbreviation is sometimes used for two different OMV types. Different induction methods will result in different OMVs. Therefore, OMVs should be extensively studied before being used in immunization studies. In order to compare results from different studies, a common nomenclature is useful. A suggested nomenclature is summarized in Table 1 for all OMV types currently described.

**Table 1:** Summary of used abbreviations for OMVs based on their induction method, as described in the text.

Method	Abbreviation	Yield	Remarks
No induction	sOMV	Low	-
Genetically induced OMVs	gOMV	Variable	Possible change in cargo
Detergent induced OMVs	dOMV	High	Loss of LPS and lipoproteins
Sonication induced OMVs	lOMV	High	Contamination with IM
Extraction molecule induced OMVs	nOMV	High	Potential loss of membrane stability
Peptide induced OMVs	pOMV	Low	Potential loss of membrane stability
Antibiotic induced OMVs	aOMV	Variable	Antibiotic presence or resistance
Heat induced OMVs	hOMV	High	Possible change in lipid composition

### Isolation of Secreted OMVs

Isolation of OMVs is independent of the induction method used and literature shows very similar procedures with small differences between studies. First, OMVs are separated from bacteria by centrifugation (Klimentová and Stulík, 2015). Next, contaminations are removed by filtration. In literature, the use of both 0.22 and 0.45 μm filters have been described, being the first discrepancy between methods. The use of a 0.22 μm filter could decrease yields by preventing passage of the larger vesicles, since vesicle sizes range between 20 and 300 nm (Kulp and Kuehn, 2010; Schwechheimer and Kuehn, 2015). After filtration, OMVs can be concentrated by precipitation or ultrafiltration (Klimentová and Stulík, 2015). Vesicles are eventually collected by ultracentrifugation, ranging from 40,000 up to 175,000x*g*, depending on the bacterial species studied. Unfortunately, rotor type and centrifugation times are not specified in most papers, although these parameters are critical for yields of OMVs (Kohl et al., 2018). Furthermore, ultracentrifugation alone may leave contaminants still present in the isolated OMV fraction. Sucrose density gradient ultracentrifugation will result in the purest fraction of OMVs and therefore also in the most consistent results between labs (Lee et al., 2016). When isolation methods are not described in detail, results obtained in immunization studies are not relevant for industrial application. To ensure possibilities to replicate experiments, transparency and detailed description of methods is critical. This will aid the scientific community and increase the relevance and comparability of described results, which could eventually accelerate OMV-based therapeutic applications.

### Applications of OMVs

The use of OMVs as a vaccine for their originating bacterium will be elaborated on below, but OMVs have many more therapeutic purposes. For instance, OMVs could also be suitable as a carrier system for proteins, glycans, and other molecules (Gerritzen et al., 2017; Gnopo et al., 2017). OMVs may be decorated with proteins, for instance, by coupling heterologous antigens to endogenous autotransporters in a hypervesiculating bacterial strain. This technique is developed for the hemoglobin-binding protease (Hbp) of *E. coli* in a hypervesiculating *Salmonella enterica* serovar typhimurium SL3261, using not only genetic engineering but also click chemistry to ensure display of larger antigens (Jong et al., 2014; van Ulsen et al., 2018). This technique can provide a robust system using well-defined OMVs as carrier that can be decorated with antigens of any bacterium of interest. The principle was demonstrated for antigens of *Mycobacterium tuberculosis* and *Chlamydia trachomatis*, where the antigens were shown to be processed and recognized (Daleke-Schermerhorn et al., 2014). Not only can protein antigens be displayed on the OMV surface, but also heterologous glycans can be displayed. Delivery of *Salmonella* O-antigen by gOMVs induced high levels of IgG antibodies in mice (De Benedetto et al., 2017). Glycosylated OMVs have also been proven to protect against subsequent bacterial challenges and may be another route of immunization with the use of OMVs (Gnopo et al., 2017). Thus, OMVs are useful as carrier system, and they also have useful intrinsic adjuvant properties (Tan et al., 2018). The presence of LPS can activate the innate immune system, thereby enhancing a subsequent immune response.

Besides using OMVs as carrier for the delivery of antigens, they could also be loaded with therapeutic molecules. *Escherichia coli* OMVs decorated with human epidermal growth factor receptor 2 (HER2) specific antibodies and loaded with siRNAs were shown to target HER2-tumor cells and exert cytotoxic effects (Gujrati et al., 2014). The advantage of using natural OMVs over synthetic liposomes is their enhanced fusion capability with target cells (Wang et al., 2018). These examples altogether show the versatile applications of OMVs and the exciting progress made over the last decades.

## OMVs in Vaccination

OMVs have been implicated in many different carrier functions, as described above. However, OMVs also have a great potential as endogenous vaccine. The presence of several antigens on OMVs limits the possibilities for pathogens to mutate all the target antigens present in the vaccine and thereby limits the possibility to generate vaccine escape variants. Furthermore, OMV isolation is relatively low-cost, compared to manufacturing of synthetic molecules for instance. This makes OMVs of great interest for vaccine development (van der Pol et al., 2015).

*In vivo*, OMVs have a wide variety of interactions with immune cells showing their potential to be used for immunization (Kaparakis-Liaskos and Ferrero, 2015; Cai et al., 2018). The first studies into immune responses evoked by OMVs already showed promising inductions of cytokines and chemokines in macrophages and other cell types. sOMVs isolated from *Brucella melitensis* were used to stimulate bone marrow-derived macrophages and showed induction of interleukin (IL)-6, IL-10, IL-12, or tumor necrosis factor (TNF) α, depending on the LPS structure of the strain used (Avila-Caldern et al., 2012). sOMVs from *E. coli* were shown to induce CXCL1 expression in mouse endothelia, leading to an increased influx of neutrophils (Lee et al., 2018). *Escherichia coli* gOMVs, loaded with a *Chlamydia muridarum* antigen, elicited a neutralizing antibody response, in contrast to recombinant antigen (Bartolini et al., 2013). This was confirmed for several other heterologous antigens loaded in *E. coli* gOMVs (Fantappiè et al., 2014), showing the benefit of retaining native conformation of antigens in OMVs.

For some bacteria, studies on immunization with OMVs in mice have been performed and showed protection against subsequent infection. For example, immunization with sOMVs from *Vibrio cholerae* in mice induced immunoglobulin production and demonstrated a protective effect toward this bacterium in their offspring (Schild et al., 2008). Studies on *E. coli* sOMVs in mice revealed that immunization with sOMVs protected against sepsis and mainly induced the protective effect *via* T cell immunity (Kim et al., 2013). For *Shigella flexneri*, merged sOMVs were used to immunize mice and also this provided protection against a subsequent lethal bacterial *Shigella* challenge (Camacho et al., 2011). An sOMV-based vaccine against *Burkholderia pseudomallei* provided protection in a mouse model and even induced humoral immunity in a nonhuman primate immunization model (Petersen et al., 2014). In chicken, an sOMV-based vaccine against *Salmonella enterica* protected against a subsequent challenge and induced high expression of interferon γ (Li et al., 2020a). All together the potential of OMVs for the use as a vaccine component seems promising. Induction and isolation methods will have consequences for immune properties of OMVs, which was shown for *Acinetobacter baumannii*. sOMVs and two types of vesicular structures prepared from the bacterial pellet were tested and while immunization with both types elicited protection against subsequent challenge, antibody profiles differed substantially (Li et al., 2020b). However, two types of OMV-based vaccine against *Neisseria meningitidis* are currently the only OMV-based vaccines licensed, MeNZB and Bexsero, and research into these will be discussed in more detail below (Oster et al., 2005; Acevedo et al., 2014; FDA, 2015).

### The Success Story of *Neisseria meningitidis*

One Gram-negative bacterium for which a safe and effective OMV-based vaccine has been in use since 1990 is *N. meningitidis* (Holst et al., 2013). This capsule forming bacterium has several serogroups and for most serogroups vaccines have been developed, except for serogroup B, which is estimated to be the cause of 65% of all meningitis cases in children under 5 years of age in the United States and 51% of total cases in Europe (Centers for Disease Control and Prevention, 2017; European Centre for Disease Prevention and Control, 2019). The vaccines for other *N. meningitidis* serogroups rely on recombinant capsular proteins, but for serogroup B the capsular protein resembles a molecule in the human brain (Finne et al., 1983). This provokes the risk of auto-immunity when used in a vaccine and therefore a different vaccine approach was necessary.

The vaccine approach for serogroup B was focused on OM proteins. To maintain stability and native fold of OM proteins, it is essential to utilize them in a membranous environment and therefore OMVs were considered as most promising for this approach. The most abundant OM protein in *N. meningitidis* OMVs was shown to be the porin protein PorA, which is also the most immunogenic protein (Granoff, 2010). Unfortunately, variation in PorA is substantial among various serogroup B strains and little cross-protection is observed (Sacchi et al., 2000). It was suggested that more than 20 different PorA molecules should be included in the vaccine to cover all *N. meningitidis* strains circulating worldwide (Sacchi et al., 2000). Therefore, no worldwide vaccine has been developed yet. However, OMV-based vaccines have proven to be very effective to control clonal outbreaks. Several outbreaks have occurred in the past, including in Cuba, Norway (Holst et al., 2009), New Zealand (Holst et al., 2013), and Normandy (Sevestre et al., 2017). Because these outbreaks were caused by a single *N. meningitidis* serogroup B strain, a dOMV vaccine was employed to prevent further spread and causalities. Analysis of the immune responses elicited by the OMV-based vaccine in Normandy demonstrated that it indeed elicited short-lasting responses, but it also elicited larger strain coverage than expected (Sevestre et al., 2017). Effectiveness of OMV-based vaccines was determined to be 87% after 10 months for the vaccines used in Cuba and Norway (Holst et al., 2009), and around 80% in New Zealand (Holst et al., 2013). However, these numbers are not based on clinical efficacy trials and therefore have to be assessed critically. Nevertheless, OMV-based vaccines are a safe and effective measure to control clonal epidemics of *N. meningitidis* and might even show cross-protection (Trzewikoswki de Lima et al., 2019).

OMV-based vaccines for *N. meningitidis* used in clonal outbreaks were prepared using detergent extraction, and thus removal of large amounts of LPS, decreasing the reactogenicity of the vaccine and increasing the necessity of an external adjuvant. Currently, detergent free nOMVs from *Neisseria* are being developed, using EDTA (van de Waterbeemd et al., 2010, 2012). Additionally, OMV yields have been improved by deletion of the *rmpM* gene in the bacterium (van de Waterbeemd et al., 2010). This gene codes for a peptidoglycan-binding outer membrane protein. Removal of the *rmpM* gene results in decreased attachment between the PG and OM, and thereby an increased formation of vesicles. Since OMVs were purified without detergents, no LPS was removed. To decrease toxicity of LPS, a second genetic modification has been implemented, by generating a knock-out of the *lpxL1* gene (van Der Ley et al., 2001). Mutants lacking the acyltransferase lpxL1 produce LPS containing five acyl chains as opposed to the regular six. This altered LPS results in decreased activation of toll like receptor 4 (TLR4) and is therefore less reactogenic, but it does not affect bacterial growth (van Der Ley et al., 2001; Fransen et al., 2010). This ngOMV-based vaccine of *Neisseria* has shown promising results in clinical trials and no severe adverse effects have been observed (Keiser et al., 2011). Research has even shown the possibility of a continuous production of *N. meningitidis* gOMVs, without the use of EDTA (Gerritzen et al., 2019). This example shows how OMV-based vaccines could be a promising strategy for combatting diseases caused by Gram-negative bacteria. The different types of OMVs studied for *N. meningitidis* are summarized in Table 2.

**Table 2:** Overview of tested OMV types for *Neisseria meningitidis* and *Bordetella pertussis* and their results.

Bacterium	OMV type	Modifications	Results	Remarks
*Neisseria meningitidis*	dOMVs	None	80–87% effectiveness	Clonal outbreaks
	ngOMVs	ΔrmpM, *Δ*lpxL1	79% effectiveness	41–82% cross-reactivity
*Bordetella pertussis*	lgOMVs	PagL	5-fold decrease in bacterial colonization	Compared to naïve mice
	nOMVs	None	5-fold decrease in bacterial colonization	Compared to naïve mice
	sOMVs	None	5-fold decrease in bacterial colonization	Compared to naïve mice at day 63

*Neisseria meningitidis* OMVs results are obtained in humans (Holst et al., 2009, 2013; Keiser et al., 2011), *Bordetella pertussis* OMV results are obtained from mice experiments (Roberts et al., 2008; Asensio et al., 2011; Raeven et al., 2016).

### OMV-Vaccine Candidate: *Bordetella pertussis*

*Bordetella pertussis* is a Gram-negative bacterium for which an OMV-based vaccine might be the optimal strategy for disease prevention. The bacterium is the causative agent for pertussis, or whooping cough, a disease most dangerous for infants (Cherry, 2016). Upon inhalation or ingestion of the bacterium, it adheres to ciliated cells and invades the lungs (Kilgore et al., 2016). Because *B. pertussis* attaches to and immobilizes the cilia, the infected individual cannot clear debris from the lungs and develops coughing fits. This results in the risk of suffocation, particularly in infants (Melvin et al., 2014).

Due to the severity of *B. pertussis* infection and the mortality caused in infants, vaccines were developed as soon as the causative agent of pertussis was identified in 1906 by Jules Bordet and Octave Gengou. The first pertussis vaccine was licensed in 1914 and consisted of whole-cell inactivated bacteria (Ligon, 1998). This whole-cell pertussis vaccine (wPv) provided satisfactory efficacy but due to adverse effects of the vaccine, like systemic fever, convulsions and even acute encephalopathy, most countries switched in the 1990s to an acellular pertussis vaccine (aPv). aPv contains 3–5 purified *B. pertussis* proteins and does not elicit adverse effects. However, aPv has shown waning immunity, partly because *B. pertussis* mutates vaccine antigens such as pertactin (Barkoff et al., 2019; Jayasundara et al., 2020). Additionally, the aPV does not evoke the effective T helper 1 cell (Th1)/T helper 17 cell (Th17) response that a natural infection evokes in humans, but a T helper 2 cell (Th2) response (Burdin et al., 2017). Furthermore, the current vaccine can prevent disease but not transmission as shown by studies in a baboon model (Pinto and Merkel, 2017). By this, *B. pertussis* can maintain itself in a population, causing disease in non-vaccinated individuals, such as infants.

The incidences of *B. pertussis* infections are increasing, despite high-vaccination coverage. Worldwide approximately 140,000 cases were reported in 2016, despite the vaccination coverage of approximately 90% (WHO, 2016; CDC, 2017). This increase in the number of cases was observed around the same time the vaccination program for *B. pertussis* was changed in the 1990s. Therefore, development of an increased immunogenic *B. pertussis* vaccine that can elicit the right immunological response and maintain increased immunological memory has become a priority (Rumbo and Hozbor, 2014; Brennan, 2017; Debrie et al., 2019). Recently, the optimal administration of a *B. pertussis* vaccine was investigated in mouse experiments and was found to be intranasal, which might increase effectiveness of new vaccines (Raeven et al., 2018). However, experiments in baboons will give more relevant information, since their immune system is more representative of a human immune system.

*Bordetella pertussis* OMVs have been extensively studied as an alternative strategy for vaccine development, since wPv has shown adverse effects and aPv has shown waning immunity (Hozbor, 2017, 2019). *Bordetella pertussis* lOMVs have been studied first and induced using sonication methods. Additionally, the *pagL* gene was introduced in this bacterial strain, which removes one acyl chain of the LPS, to decrease LPS toxicity (therefore resulting in lgOMVs; Asensio et al., 2011). Immunization with these vesicles showed faster clearance of bacteria in the lungs of infected mice compared to non-immunized mice. Furthermore, immunization of mice with lgOMVs showed decreased gene expression of inflammatory cytokines compared to immunization with lOMVs. Previous attempts to detoxify LPS by genetic removal of acyl chains did not always lead to these results, sometimes endotoxic effects were even increased (Geurtsen et al., 2006). This is probably due to an increased LPS release upon modification, which resulted in increased TLR4 activation (Geurtsen et al., 2006). Recently, the immune response evoked by *B. pertussis* lOMVs was studied further and revealed to activate the inflammasome in mice and human macrophages (Elizagaray et al., 2020). However, since the lOMVs or lgOMVs were extracted using sonication, which disrupts the entire bacterial membrane, contamination of the OMV sample by other bacterial products could have occurred, or the loss of natural cargo, making the studied immune responses not relevant to *in vivo* produced sOMVs (Asensio et al., 2011).

In later studies, *B. pertussis* nOMVs have also been used in *in vivo* mice experiments (Roberts et al., 2008). Immunization with *B. pertussis* nOMVs, extracted by EDTA, resulted in a rapid clearance of bacteria after challenge, similar to immunization with killed whole-cell *B. pertussis*. Characterization of *B. pertussis* nOMVs revealed that the presence of pertussis toxin and pertactin in the nOMVs is essential for evoking an effective immune response (Ormazábal et al., 2014). *Bordetella pertussis* nOMVs elicited a long-lasting protection, for up to 9 months in mice (Gaillard et al., 2014). However, it is unsure how this can be translated to humans.

More recently, *B. pertussis* sOMVs have been used to study the immune response. The adaptive immune responses evoked by these sOMVs have been characterized extensively in mice. Both immunization with sOMVs and heat-killed whole-cell *B. pertussis* evoked mixed Th1/Th2/Th17 responses but the sOMV-based vaccine seems to induce a different antibody response. After booster immunization, the antibody profile was dominated by IgG3 for the sOMV-based vaccine and IgG1 for the whole-cell based vaccine (Raeven et al., 2016). The most prominent antibody response was shown to be directed against BrkA, Vag8, and LOS, all outer membrane components (Raeven et al., 2015). Most importantly, the sOMV-based vaccine showed less pro-inflammatory cytokine production compared to the whole-cell vaccine (Raeven et al., 2016). This suggests that a sOMV-based vaccine could resolve any reactogenicity problems encountered by the whole-cell vaccine. All types of studied *B. pertussis* OMVs are summarized in Table 2.

## Future Prospects

OMV-based vaccines have great potential for next generation vaccine development. Several challenges remain, such as yields of OMVs, after isolation and the composition and thereby immunogenicity and toxicity of the vesicles (van der Pol et al., 2015). While OMVs are a natural product and beneficial to the bacterium, no large quantities are produced during bacterial growth but there might be a rather simple solution to increase OMV yields. OMV release has been shown to increase upon stress, as described above. The most trivial stress a bacterium could experience is environmental stress, for instance, nutrient depletion, pressure, or temperature stress (van de Waterbeemd et al., 2013b). In *Pseudomonas putida*, it was shown that a heat shock of 55°C increased OMV release (von Bergen et al., 2012). Similarly, after treatment with higher temperatures B-band LPS export in OMVs was increased in *P. aeruginosa* (Eberlein et al., 2018). Recently it was shown that heat treatment also increased OMV production in *B. pertussis* (de Jonge et al., 2021). These heat-induced OMVs (hOMVs) were shown to still contain important antigens, which could be detected with antibodies. Furthermore, the same treatment was applied to *B. bronchiseptica* and the resulting OMVs were further characterized to ensure quality of the vesicles. hOMVs were stable up to 40°C and sOMVs even up to 50°C. Additionally, hOMVs had a large increase in the amount of lysophospholipids, as was shown by lipidomic analysis. Despite these differences, hOMVs evoked a comparable immune response to spontaneous OMVs *in vitro* (Balhuizen et al., 2021). However, the quantities of LPS might still pose a problem and molecules to modulate the resulting immune response are needed.

AMPs were originally known for their antimicrobial function, but recently immunomodulatory functions have been described for these peptides as well (Hilchie et al., 2013; Hancock et al., 2016; van Harten et al., 2018; Mookherjee et al., 2020; Scheenstra et al., 2020). For example, the human cathelicidin antimicrobial peptide LL-37 has been shown to direct dendritic cell (DC) differentiation to promote a Th1 response (Davidson et al., 2004). This could be employed in vaccine development by steering the immune response to a desired Th1/Th17 response. Furthermore, the chicken cathelicidin 2 (CATH-2) was shown to induce several chemokines, suggesting that immunomodulatory mechanisms might be conserved among species (van Dijk et al., 2016; van Harten et al., 2018). On the other hand, LL-37 has also been shown to inhibit TLR4 activation on DCs by agonists such as LPS (Kandler et al., 2006). Likewise, CATH-2 was shown to neutralize LPS-induced TLR4 activation by interacting with LPS. This was shown in the context of non-viable bacteria, possibly as a mechanism to prevent an unnecessary immune response (Coorens et al., 2017; Scheenstra et al., 2020). Therefore, AMPs could decrease LPS-induced TLR4 activation in an OMV-based vaccine, as was recently been shown for *B. bronchiseptica* OMVs. When the porcine AMP, PMAP-36, was supplemented to isolated sOMVs and subsequently used to stimulate macrophages, cytokine secretion decreased (Balhuizen et al., 2021). Furthermore, a synthetic anti-endotoxin (non-AMP) peptide was also shown to decrease *E. coli* OMV-induced activation of human macrophages (Pfalzgraff et al., 2019). These results indicate that AMPs are the promising molecules for tailoring immune responses in vaccines, however, studies on other pathogens should reveal whether this mechanism is broadly applicable. Furthermore, tailor-made AMPs could be synthesized with desired immune modulating properties (Haney et al., 2017; Wuerth et al., 2017; Etayash et al., 2020).

## Conclusion

OMVs are a promising tool for vaccine development, especially compared to acellular vaccines. The immunogenicity of OMV based vaccines is increased compared to acellular vaccines and the risk of evolutionary escape pathogens is almost diminished compared to using an acellular vaccine. Especially in cases where whole-cell approaches are not applicable, OMV-based vaccines pose a potential solution. However, some challenges lie ahead of the OMV-based vaccine field such as low yields and endotoxic effects due to the presence of LPS. Many solutions have been created such as extraction to increase vesicle yields or genetic modifications to both increase yields and decrease endotoxicity. However, these solutions often alter vesicles as such that their representation of the originating bacterium is no longer optimal. The use of spontaneous OMVs would circumvent this. To increase yields of sOMVs, a simple solution seems to be optimal: heat induction. To reduce LPS endotoxicity, host defense peptides show great potential. These peptides are known for their antimicrobial activity but additionally have shown to exhibit immunomodulatory activities such as the neutralization of LPS-induced TLR4 activation. Furthermore, they can steer immune responses, possibly into an ideal Th1/Th17 response. Concluding, induced OMVs are a promising future for bacterial vaccine development, with AMPs being a potential solution to the challenges that lie ahead.

## Author Contributions

MB designed the study. MB, EV, and HH wrote the manuscript. All authors contributed to the article and approved the submitted version.

### Conflict of Interest

The authors declare that the research was conducted in the absence of any commercial or financial relationships that could be construed as a potential conflict of interest.
